# Benzylidene-bis-(4-Hydroxycoumarin) and Benzopyrano-Coumarin Derivatives: Synthesis, ^1^H/^13^C-NMR Conformational and X-ray Crystal Structure Studies and *In Vitro* Antiviral Activity Evaluations

**DOI:** 10.3390/molecules16076023

**Published:** 2011-07-19

**Authors:** Davorka Završnik, Samija Muratović, Damjan Makuc, Janez Plavec, Mario Cetina, Ante Nagl, Erik De Clercq, Jan Balzarini, Mladen Mintas

**Affiliations:** 1 Faculty of Pharmacy, University of Sarajevo, Čekaluša 90, Sarajevo BA-71000, Bosnia and Hercegovina; 2 Slovenian NMR Centre, National Institute of Chemistry, Hajdrihova 19, Ljubljana SI-1000, Slovenia; 3 EN-FIST Centre of Excellence, Dunajska 156, Ljubljana SI-1000, Slovenia; 4 Faculty of Chemistry and Chemical Technology, University of Ljubljana, Aškerčeva cesta 5, Ljubljana SI-1000, Slovenia; 5 Department of Applied Chemistry, Faculty of Textile Technology, University of Zagreb, Prilaz baruna Filipovića 28a, Zagreb HR-10000, Croatia; 6 Rega Institute for Medical Research, Katholieke Universiteit Leuven, Minderbroedersstraat 10, Leuven B-3000, Belgium; 7 Department of Organic Chemistry, Faculty of Chemical Engineering and Technology, University of Zagreb, Marulićev trg 20, Zagreb HR-10000, Croatia

**Keywords:** 4-hydroxycoumarin, benzopyranocoumarin, antiviral activity, ^1^H/^13^C-NMR conformational study, X-ray diffraction

## Abstract

We report on the synthesis of 4-hydroxycoumarin dimers **1**–**15** bearing an aryl substituent on the central linker and fused benzopyranocoumarin derivatives **16**–**20** and on their *in vitro* broad anti-DNA and RNA virus activity evaluations. The chemical identities and structure of compounds **1**–**20** were deduced from their homo- and heteronuclear NMR measurements whereas the conformational properties of **5**, **14** and **20** were assessed by the use of 1D difference NOE enhancements. Unequivocal proof of the stereostructure of compounds **7**, **9**, **16** and **18** was obtained by single crystal X-ray diffraction method. The X-ray crystal structure analysis revealed that two 4-hydroxycoumarin moieties in the 4-trifluoromethylphenyl- and 2-nitrophenyl derivatives (compounds **7** and **9**, respectively) are intramolecularly hydrogen-bonded between hydroxyl and carbonyl oxygen atoms. Consequently, the compounds **7** and **9** adopt conformations in which two 4-hydroxy-coumarin moieties are *anti*-disposed. Antiviral activity evaluation results indicated that the 4-bromobenzylidene derivative of bis-(4-hydroxycoumarin) (compound **3**) possesses inhibitory activity against HSV-1 (KOS), HSV-2 (G), vaccinia virus and HSV-1 TK^-^ KOS (ACV^r^) at a concentration of 9–12 μM and at a minimum cytotoxic concentration (MCC) greater than 20 μM. Compounds **4**–**6**, **8**, and **20** were active against feline herpes virus (50% effective concentration, EC_50_ = 5–8.1 μM), that is at a 4-7-fold lower concentration than the MCC.

## 1. Introduction

Numerous experimental studies indicate that natural and synthetic coumarins (2*H*-1-benzopyran-2-ones) and their derivatives are endowed with excellent chemical reactivity and different bioactivity. Thus, the natural coumarins play an important role in plant biochemistry and physiology. They act as antioxydants, enzyme inhibitors and precursors of toxic substances. They are also involved in the actions of plant growth hormones and growth regulators, the control over the respiration and photosynthesis, as well as in the defense against various infections [1]. Although most of the existing natural coumarins have been isolated from higher plants, some of them have been discovered in microorganisms, e.g., aminocoumarin antibiotics: Novobiocin, coumermycin A_1_ and chlorobiocin (produced by the actinomycete *Streptomyces niveus*) [2].

Synthetic coumarin derivatives have been obtained by chemical modification of the coumarin ring. As a substitution can conceptually occur at any of the six available sites of the basic molecule, these compounds are widely variable in structure and activity. The biological activities of coumarin derivatives, in particular their therapeutic application as anticoagulant and antibacterial agents [3], has stimulated further interest for the synthesis of this class of compounds. A variety of synthesized coumarin derivatives have been experimentally shown to exert pharmacological activities including inhibition of platelet aggregation, cytochrome P450, and steroid 5-α-reductase.

**Figure 1:**
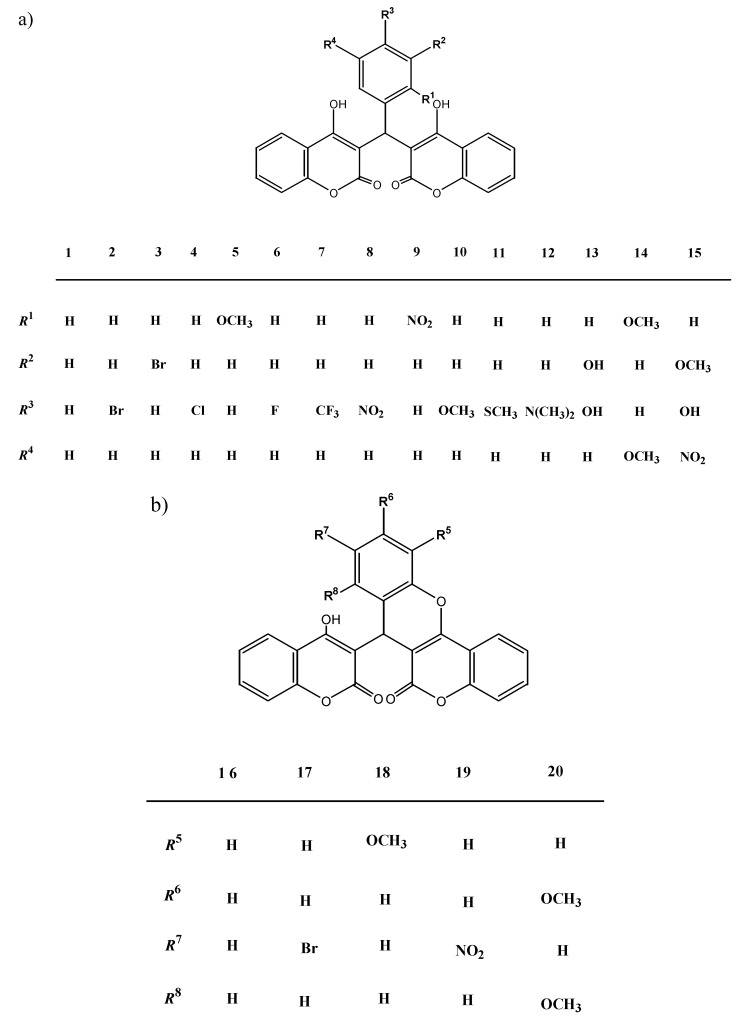
(**a**) The benzylidene-bis-(4-hydroxycoumarin) derivatives **1**–**15**; (**b**) 3-[6-oxo-(1H)-benzopyrano[4,3-b]benzopyran-7-yl]-4-hydroxycoumarin derivatives **16**–**20**.

They have also been shown to exert efficient anti-proliferative, antifungal, anti-psoriasis, anti-inflammatory, as well as antiviral activities [4,5,6,7,8]. The interest in coumarins has recently increased significantly because it was found that they inhibit HIV (human immunodeficiency virus), by affecting integrase and reverse transcriptase, which play a critical role in the replicative cycle of HIV [9,10,11]. The present study is focused on the antiviral activity evaluation of the benzylidene-bis-(4-hydroxycoumarin) derivatives **1**–**15** and 3-[6-oxo-(1*H*)-benzopyrano[4,3-b]benzopyran-7-yl]-4-hydroxycoumarin derivatives **16**–**20** (Figure 1).

## 2. Results and Discussion

### 2.1. Chemistry

**Scheme 1:**
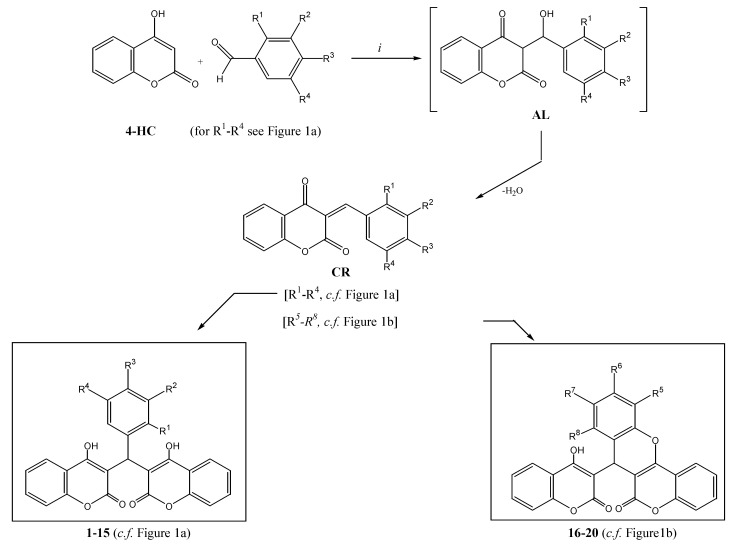
Synthesis of coumarin-dimer derivatives with variously substituted aryl central linkers (**1**–**15**) and fused benzopyranocoumarin derivatives **16**–**20**. Reagents and conditions: (*i*) EtOH, heating under reflux for 24 h.

The benzylidene-bis-(4-hydroxycoumarin) derivatives **1**–**15** and fused benzopyranocoumarin derivatives **16**–**20** were prepared by a sequence of reactions displayed in the Scheme 1. In the first step of the synthesis the aldol condensation of 4-hydroxycoumarin (**4-HC**) with an appropriately substituted aldehyde linker followed by dehydration of aldol product (**AL**) gave a chromone (**CR**). Subsequent *in situ* reaction of the chromon with 4-hydroxycoumarin already present in excess in the reaction mixture gave dimeric coumarin derivatives **1**–**15** bearing an aryl substituent in the central methylene linker. In contrast, the chromone derivatives containing an *ortho*-substituted phenyl moiety (R^1^ = Cl, F, OH, OCH_3_) gave under spontaneous cyclization the fused benzopyranocoumarin derivatives **16**–**20**. The compounds **1** [11,14,16], **2** [11,13,14], **4** [14], **5** [14], **6** [13], **8** [14], **9** [11,14], **10** [14], **11** [14], **12** [11,14] and **16** [17,18] that have been described previously were also synthesized for their antiviral activity evaluations.

### 2.2. NMR Assignment and Conformational Study

The chemical identities and structures of **1**–**20** were confirmed by homo- and heteronuclear NMR measurements. ^1^H-, ^13^C- and ^19^F-NMR chemical shifts are reported in the Experimental section. Proton-decoupled ^13^C-NMR spectra showed C–F coupling constants that enabled straightforward identification of fluorinated carbon atoms and their neighbors. In the ^1^H spectrum of **1**, **2**, **4** and **10**, which were dissolved in non-polar solvents, well resolved hydroxyl protons were observed in the range from δ 11.3 to 11.5 ppm (see Experimental section for details). The strongly deshielded signals suggest that OH protons are most probably involved in hydrogen bond formation. In addition, two set of ^1^H-NMR signals were observed for H5 and H5” protons which indicates a slight difference in magnetic environment for otherwise symmetrical moieties. Furthermore, hydroxyl protons, which were observed between δ 11.8 and 12.3 ppm for benzopyranocoumarin derivatives **16**, **18** and **20**, are most likely involved in hydrogen bonds.

**Figure 2:**
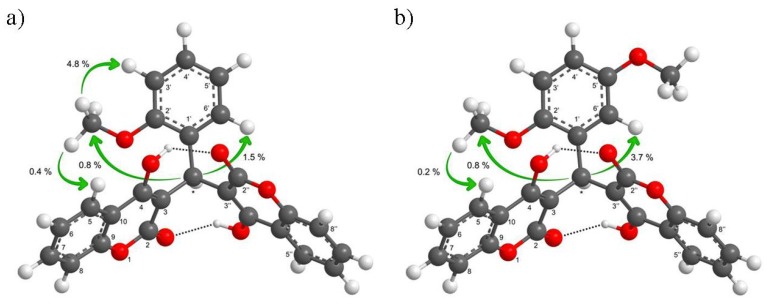
Key NOE enhancements in **5** (**a**) and **14** (**b**). Dotted lines present hydrogen bonds.

Hydrogen bonds described above partially determined conformational preferences of the studied compounds. Conformational properties of **5**, **14** and **20** were assessed with the use of 1D NOE difference experiments. Key NOE enhancements are shown in Figure 2. The saturation of H* in **5** resulted in weak NOE at H6′ (1.5%) and 2′-OCH_3_ (0.8%, Figure 2a).

**Figure 3:**
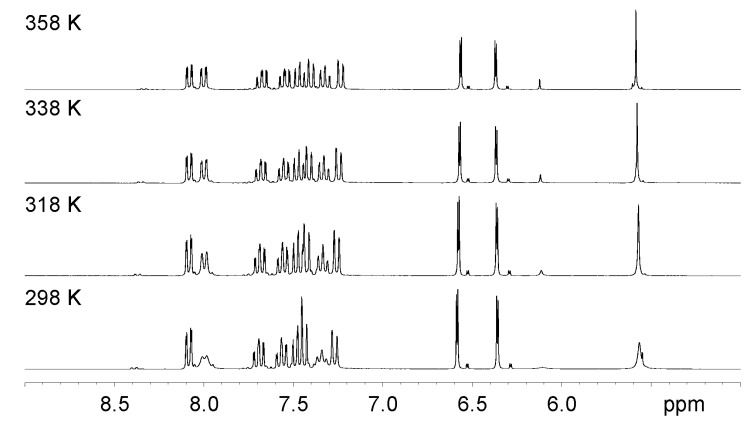
^1^H-NMR spectra of **20** dissolved in DMSO-d6 at temperatures in the range from 298 to 358 K.

Likewise, the saturation of H* in **14** gave moderate NOE at H6′ (3.7%) and weak NOE at 2′-OCH_3_ (0.8%). These observations suggested nonrestricted rotation along C*–C1′ bonds in **5** and **14**. No NOEs indicative of relative orientations of individual heterocyclic moieties were observed for **20**. Interestingly, benzopyranocoumarin derivatives **16**, **18** and **20** showed two sets of signals in the ^1^H spectrum for coumarin protons. One set of ^1^H signals (H5-H8 protons) exhibited broader line-widths with respect to multiplets attributed to H5”-H8” protons. This phenomenon was studied in more detail for **20** by variable temperature experiments in the range from 298 to 358 K. As an example, broad multiplet at δ 8.00 ppm corresponding to H5 becomes sharper at higher temperatures (Figure 3). This suggests that rotation along C*–C3 bond is restricted at lower temperatures, most likely due to formation of hydrogen bond between the C4-OH and C2” carbonyl group (for enumeration of atoms *c.f.* Figure 2).

### 2.3. X-Ray Crystal Structure Study

**Figure 4:**
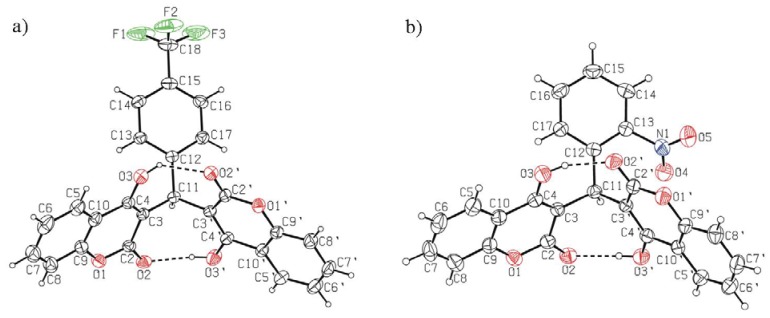
A view of **7** (**a**) and **9** (**b**), with the atom-numbering scheme. Displacement ellipsoids for non-hydrogen atoms are drawn at the 20% and 30% probability level for **7** and **9**, respectively. Intramolecular hydrogen bonds are indicated by dashed lines.

In **7** (Figure 4a) and **9** (Figure 4b), two 4-hydroxycoumarin moieties are linked through a methylene bridge on which one hydrogen atom has been replaced with a phenyl ring bearing *p*-trifluoromethyl and *o*-nitro groups, respectively. In general, the geometry of the molecules agrees with closely related structures [19,20,21].

The exocyclic bond angles around C3 atom in **7** and **9** differ by even 11.7^o^ and 11.6^o^, respectively. The corresponding angles about C3′ atom of the coumarin ring differ by only 5.6^o^ in **7** and 4.0^o^ in **9**. This asymmetry, which is also found in similar structures, may be a consequence of steric crowding within the molecules. Because of the same reason all principal bond angles about C11 are widened over normal tetrahedral values, ranging from 112.5(2) to 115.4(2)° in **7** and 111.75(12) to 115.63(12)° in **9**. The coumarin rings are slightly distorted from planarity, with two planes inclined at 57.83(11)° and 59.55(5)° to each other in **7** and **9**, respectively.

The 4-hydroxycoumarin moieties are intramolecularly hydrogen bonded between hydroxyl and carbonyl oxygen atoms in both structures (see Figure 4a and Figure 4b and Table S1 in ESI), thus forming two eight-membered rings. Thus, compounds **7** and **9** adopt a conformation in which two 4-hydroxycoumarin moieties are anti-disposed. Benzylidene bis (4-hydroxycoumarin) derivatives **7** and **9** form supramolecular self-assemblies by C−H···O hydrogen bonds (Figures S1 and S2, see ESI) in which infinite chains are extended by one F···F interaction between trifluoromethyl fluorine atoms in **7** [F2···F2*^i^* = 2.934(5) Å; (*i*): −x, −y, −z] and one π···π interaction in **9**. The distance between the ring centroids of coplanar C5′–C10′ rings [*α* = 0^o^] in **9** is 3.6832(12) Å, the planes are separated by 3.5132(8) Å and centroids offset is *ca*. 1.11 Å.

**Figure 5:**
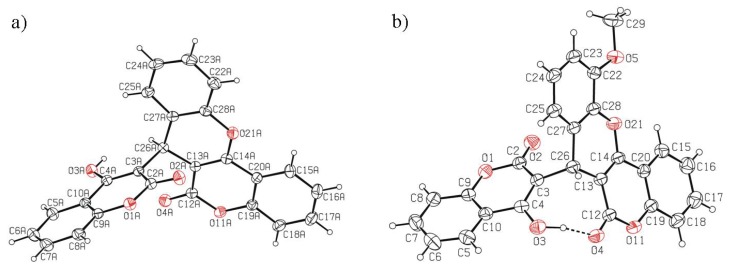
A view of **16** (**a**) and **18** (**b**), with the atom-numbering scheme. For clarity, only one independent molecule of **16** is shown. Displacement ellipsoids for non-hydrogen atoms are drawn at the 30% probability level. Intramolecular hydrogen bond in 18 is indicated by dashed line.

Cyclized compound **16** (Figure 5a) crystallized with two independent molecules, two ethanol molecules and one water molecule in the asymmetric unit in monoclinic space group *P* 2_1_/*c*. The bond lengths in two independent molecules of **16**, denoted as A and B, are within 2*σ* values, and agree very well with the corresponding ones in **18** (Figure 5b).

The main structural difference between these two structures is in orientation of the coumarin rings. The coumarin ring in **18** is flipped by approximately 180^o^ compared to the situation in **16**, and such ring orientation enables O−H···O intramolecular hydrogen bond formation (Table S1, see ESI). Two independent molecules of **16**, two ethanol molecules and water molecule are linked by six O−H···O and four C−H···O hydrogen bonds into three-dimensional network (Figure S3, see ESI). Two C−H···π interactions participate also in supramolecular aggregation. On the contrary, only one hydrogen bond of C−H···O type links the molecules of **18**, thus forming discrete centrosymmetric dimers *via* 20-membered rings (Figure S4, see ESI).

### 2.4. Biological Activity Results

*Antiviral activity*.-Compounds **1**–**20** were evaluated for their inhibitory activities against herpes simplex virus type 1 and 2 [HSV-1 (KOS), HSV-2 (G)], vaccinia virus, vesicular stomatitis virus and herpes simplex virus-1 TK^−^ KOS (ACV^r^) in HEL cell cultures. Their activities were compared with those of brivudin [(*E*)-5-(2-bromovinyl)-2’-deoxyuridine], cidofovir [(*S*)-1-[3-hydroxy-2-(phosphonyl-methoxy)propyl]cytosine], acyclovir [9-(3-hydroxyethoxymethyl)guanine] and gancyclovir [9-[(1,3-dihydroxy-2-propoxy)methyl]guanine] (Table 1, only data for the selected number of compounds **1**, **4**–**6**, **8**, **9** and **20** are shown).

Of all evaluated compounds only the 3-bromobenzylidene derivative **3** of bis(4-hydroxycoumarin) showed potentially interesting inhibitory activity against HSV-1 (KOS), HSV-2 (G), vaccinia virus and HSV-1 TK^−^ KOS (ACV^r^) in the range of 9–12 μM at a minimum cytotoxic concentration (MCC) greater than 20 μM, whereas compound **19** showed only slight activity against HSV-1 (KOS), HSV-2 (G) and HSV-1 TK^−^ KOS (EC_50_ = 45–50 μM) in human embryonic lung HEL cell cultures and no cytotostatic activity at 100 µM (Table 1, data taken together from two independent experiments). Evaluation of compounds **1**–**20** on Feline corona virus (FIPV) and Feline herpes virus activity and cytotoxicity in Crandell-Rees feline kidney (CRFK) cell cultures showed that the benzylidene-bis-(4-hydroxycoumarin) derivatives **1**, **4**–**6**, **8** and **9**, and the benzopyranocoumarin derivative **20** exerted *anti*-FIPV activity (EC_50_ = 7.2–10 μM) in CRFK cell cultures. Such activity is within the same magnitude as that of the plant lectin *Urtica dioica agglutinin* (UDA) [10] (EC_50_ = 6 μM) but less active than *Hyppeastrum hybride agglutinin* (HHA) [12] (EC_50_ = 0.38 μM). However it should be noted that the novel compounds showed cytostatic activities at concentrations that are only 4-5-fold higher than the antiviral active concentrations and therefore, it can be questioned whether the antiviral effect observed represents a direct antiviral effect or elicited indirectly by the cytotoxic activity of the test compounds. Similarly, the compounds **4**–**6**, **8** and **20** exhibited *anti*-Feline Herpes Virus activity (EC_50_ = 5–8.1 μM) the MCC were only 4-7-fold higher than the EC_50_ values (Table 2, only selected data for compounds **1**, **4**–**6**, **7**, **8** and **20** are shown).

Compounds **1**–**20** were also evaluated for their cytotoxicity and antiviral activities against Coxsackie virus B4 and respiratory syncytial virus (RSV) in HEL cell cultures; parainfluenza-3 virus, reovirus-1, Sindbis virus, Coxsackie virus B4 and Punta Toro virus in Vero cell cultures; and influenza virus subtypes A H1N1 and H3N2, and influenza virus B. No specific antiviral effects (*i.e*., antivirally effective concentration >5-fold lower than the minimal cytotoxic concentration) were noted for any of the evaluated compounds.

## 3. Experimental

### 3.1. General Methods

Melting points (uncorrected) were determined with Büchi melting point B-545. Precoated Merck silica gel 60F-254 plates were used for thin-layer chromatography (TLC) and the spots were detected under UV light (254 nm). Solvent system used for TLC was chloroform:methanol = 9:1. Mass spectra were recorded with an Autospec: ESI/Q-TOF Premier instrument. ^1^H-NMR spectra were recorded at 600 MHz, in deuterated DMSO-d6, on Bruker (UXNMR/XwinNMR) spectrometer, using tetramethylsilane (TMS) as internal reference. 1D and 2D NMR spectra were recorded at 25 °C on Varian Unity Inova 300 spectrometers. ^1^H and ^13^C chemical shifts were referred to residual signal of CDCl_3_, CD_2_Cl_2_ and DMSO-d_6_ (δ_TMS_ 0.0 ppm). ^19^F chemical shifts were referenced externally with respect to CCl_3_F (δ 0.0 ppm). ^1^H, ^13^C and ^19^F-NMR resonances were assigned on the basis of signal intensities and multiplicities in 1D spectra as well as correlation signals in 2D ^1^H-^1^H COSY, ^1^H-^13^C HSQC and ^1^H-^13^C HMBC NMR spectra. Elemental analyses were performed in the Central Analytic Service, Rudjer Bošković Institute Zagreb, Croatia, using a Perkin Elmer 2400 Elemental Analyser. The infrared spectra were obtained from potassium bromide triturate containing 0.5% of the product on a Perkin-Elmer 1000 FT-IR spectrophotometer. All data were recorded at 25 °C unless specified otherwise.

### 3.2. Procedures for the Preparation of Compounds

#### 3.2.1. The compounds 1–20 were prepared by the following general procedure

4-Hydroxycoumarin (2 mmol) was dissolved in hot ethanol (6 mL), the corresponding aldehyde (1 mmol) was added, and the reaction mixture was refluxed for 24 h. After cooling to room temperature, the solid was filtered off and crystallized to give the product benzylidene-bis-(4-hydroxycoumarin) derivatives **1**–**15** and 3-[6-oxo-(1*H*)-benzopyrano[4,3-b]benzopyran-7-yl]-4-hydroxycoumarin derivatives **16**–**20** (Figure 1).

#### 3.2.2. Compound data

*3,3′-(Benzilidene)-bis-[4-hydroxycoumarin]* (**1**) [11,14,16]. Yield 77.5%; mp 233.3 °C; IR (KBr) (ν, cm^−1^): 2901 (CH); 1658 (C=O); 1600, 1584, 1497, 1450 (C=C ar.); 1199 (OH); ^1^H-NMR (CDCl_3_): δ 6.11 (H*, s, 1H), 7.23 (H2′/H6′, m, 2H), 7.27 (H4′, m, 1H), 7.33 (H3′/H5′, m, 2H), 7.38 (H6/H6", m, 2H), 7.42 (H8/H8", m, 2H), 7.63 (H7/H7", m, 2H), 8.00 (H5/H5", d, *J* = 7.3, 1H), 8.08 ppm (H5/H5", d, *J* = 6.8, 1H), 11.41 (OH, b, 2H); ^13^C-NMR (CDCl_3_): δ 36.16 (C*), 103.90/105.63 (C3/C3"), 116.63 (C8/C8"), 116.91 (C10/C10"), 124.39 (C5/C5"), 124.88 (C6/C6"), 126.47 (C2′/C6′), 126.87 (C4′), 128.63 (C3′/C5′), 132.85 (C7/C7"), 135.17 (C1′), 152.29/152.53 (C9/C9"), 164.60/165.80 (C4/C4"), 166.87/169.31 ppm (C2/C2"); ES-MS *m/z* 413 [M−H].

*3,3′-(4-Bromobenzylidene)-bis-[4-hydroxycoumarin]* (**2**) [11,13,14]. Yield 74%; mp 267.2 °C; IR (KBr) (ν, cm^−1^): 3276 (OH), 2730 (CH), 1772 (C=O), 1668 (C=C–C=O), 1604, 1564, 1488, 1454 (C=C), 646 (C–Br); ^1^H-NMR (CDCl_3_): δ 6.02 (H*, s, 1H), 7.10 (H2′/H6′, m, 2H), 7.38 (H6/H6", m, 2H), 7.41 (H8/H8", m, 2H), 7.44 (H3′/H5′, m, 2H), 7.64 (H7/H7", m, 2H), 7.99 (H5/H5", d, *J* = 7.7, 1H), 8.07 (H5/H5", d, *J* = 7.3, 1H), 11.32 ppm (OH, s, 1H), 11.54 (OH, s, 1H); ^13^C-NMR (CDCl_3_): δ 35.87 (C*), 103.62/105.18 (C3/C3"), 116.33/116.67 (C10/C10"), 116.69 (C8/C8"), 120.80 (C4′), 124.41 (C5/C5"), 125.00 (C6/C6"), 128.33 (C2′/C6′), 131.70 (C3′/C5′), 133.04 (C7/C7"), 134.41 (C1′), 152.28/152.53 (C9/C9"), 164.64/166.03 (C4/C4"), 166.84/169.21 ppm (C2/C2"); ES-MS *m/z* 491 [M−H]. Elemental analysis. Calc. for C_25_H_15_O_6_Br: C 61.12, H 3.08, Found: C 61.07, H 3.13.

*3,3′-(3-Bromobenzylidene)-bis-[4-hydroxycoumarin]* (**3**). Yield 83%; mp 287 °C; IR (KBr) (ν, cm^−1^): 2918 (CH), 1774 (C=O), 1648 (C=C–C=O), 1610, 1568, 1498, 1452 (C=C), 1196 (OH), 970 (C–Br); ^1^H-NMR (DMSO-d_6_): δ 6.31 (H*, d, *J* = 0.9, 1H), 7.14 (H6′, m, 1H), 7.18 (H5′, m, 1H), 7.26 (H2′, m, 1H), 7.29 (H6/H6", m, 2H), 7.32 (H4′, m, 1H), 7.33 (H8/H8", m, 2H), 7.57 (H7/H7", m, 2H), 7.88 ppm (H5/H5", dd, *J* = 7.9, 1.7, 2H); ^13^C-NMR (DMSO-d_6_): δ 36.02 (C*), 103.32 (C3/C3"), 115.80 (C8/C8"), 118.75 (C10/C10"), 121.46 (C3′), 123.41 (C6/C6"), 124.03 (C5/C5"), 126.00 (C6′), 128.23 (C4′), 129.26 (C2′), 130.12 (C5′), 131.58 (C7/C7"), 144.40 (C1′), 152.37 (C9/C9"), 164.49 (C2/C2"), 166.51 ppm (C4/C4"); ES-MS *m/z* 491 [M−H]. Elemental analysis. Calc. for C_25_H_15_O_6_Br: C 61.12, H 3.08, Found: C 61.24, H 3.11.

*3,3’-(4-Chlorobenzylidene)-bis-[4-hydroxycoumarin]* (**4**) [14]. Yield 82%; mp 256.7 °C; IR (KBr) (ν, cm^−1^): 2858 (CH), 1828 (C=O), 1668 (C=C–C=O), 1604, 1562, 1490, 1454 (C=C), 1182 (OH), 706 (C–Cl); ^1^H-NMR (CDCl_3_): δ 6.04 (H*, s, 1H), 7.16 (H2′/H6′, m, 2H), 7.29 (H3′/H5′, m, 2H), 7.39 (H6/H6", m, 2H), 7.41 (H8/H8", m, 2H), 7.64 (H7/H7", m, 2H), 8.00 (H5/H5", d, *J* = 7.7, 1H), 8.07 (H5/H5", d, *J* = 7.7, 1H), 11.32 (OH, b, 1H), 11.54 ppm (OH, b, 1H); ^13^C-NMR (CDCl_3_): δ 35.80 (C*), 103.69/105.25 (C3/C3"), 116.34/116.81 (C10/C10"), 116.67 (C8/C8"), 124.42 (C5/C5"), 124.99 (C6/C6"), 127.97 (C2′/C6′), 128.77 (C3′/C5′), 132.73 (C4′), 133.04 (C7/C7"), 133.84 (C1′), 152.28/152.53 (C9/C9"), 164.65/166.01 (C4/C4"), 166.85/169.21 ppm (C2/C2"); ES-MS *m/z* 447 [M−H]. Elemental analysis. Calc. for C_25_H_15_O_6_Cl: C 67.19, H 3.38, Found: C 67.16, H 3.49.

*3,3′-(2-Methoxybenzylidene)bis[4-hydroxycoumarin]* (**5**) [14]. Yield 82%; mp 254.7 °C; IR (KBr) (ν, cm^−1^): 3030 (C–CH); 2898 (CH); 1670 (C=C–C=O), 1604, 1566, 1498, 1450 (C=C), 1192 (OH), 660 (C–Cl); ^1^H-NMR (DMSO-d_6_): δ 3.57 (OCH_3_, s, 3H), 6.25 (H*, s, 1H), 6.84 (H5′, m, 1H), 6.89 (H3′, d, *J* = 7.9, 1H), 7.16 (H6′, m, 1H), 7.17 (H4′, m, 1H), 7.32 (H6/H6", m, 2H), 7.36 (H8/H8", d, *J* = 8.0, 2H), 7.58 (H7/H7", m, 2H), 7.90 ppm (H5/H5", dd, *J* = 7.9, 1.2, 2H); ^13^C-NMR (DMSO-d_6_): δ 32.93 (C*), 55.51 (OCH_3_), 104.80 (C3/C3"), 110.94 (C3′), 115.91 (C8/C8"), 117.54 (C10/C10"), 119.85 (C5′), 123.60 (C5/C5"), 123.67 (C6/C6"), 127.29 (C4′), 128.22 (C6′), 128.29 (C1′), 131.63 (C7/C7"), 151.98 (C9/C9"), 157.34 (C2′), 163.63 (C4/C4"), 163.99 ppm (C2/C2"); ES-MS *m/z* 447 [M−H].

*3,3′-(4-Fluorobenzylidene)-bis-[4-hydroxycoumarin]* (**6**) [13]. Yield 86%; mp 214.5 °C; IR (KBr) (ν, cm^−1^): 2858 (CH), 1774 (C=O), 1675 (C=C–C=O), 1608, 1534, 1508, 1454 (C=C), 1188 (OH), 1030 (C–F); ^1^H-NMR (DMSO-d_6_): δ 6.31 (H*, m, 1H), 7.03 (H3′/H5′, m, 2H), 7.17 (H2′/H6′, m, 2H), 7.31 (H6/H6", m, 2H), 7.35 (H8/H8", m, 2H), 7.58 (H7/H7", m, 2H), 7.89 ppm (H5/H5", dd, *J* = 7.9, 1.7, 2H); ^13^C-NMR (DMSO-d_6_): δ 35.48 (C*), 104.01 (C3/C3"), 114.58 (C3′/C5′, d, *J_CF_* = 20.9), 115.88 (C8/C8"), 118.23 (C10/C10"), 123.59 (C6/C6"), 123.93 (C5/C5"), 128.52 (C2′/C6′, d, *J_CF_* = 7.7), 131.76 (C7/C7"), 136.34 (C1′, d, *J_CF_* = 2.8), 152.26 (C9/C9"), 160.42 (C4′, d, *J_CF_* = 241.2), 164.66 (C2/C2"), 165.68 ppm (C4/C4"). ^19^F-NMR (DMSO-d_6_): δ –117.92 ppm (C4′-F, m); ES-MS *m/z* 431 [M−H].

*3,3′-(4-Trifluoromethylbenzylidene)-bis-[4-hydroxycoumarin]* (**7**). Yield 72%; mp 271.3 °C; IR (KBr) (ν, cm^−1^): 2918 (CH), 1778 (C=O), 1652 (C=C–C=O), 1604, 1568, 1504, 1454 (C=C), 1470 (C–C), 1198 (OH), 1028 (C–F); ^1^H-NMR (DMSO-d_6_): δ 6.34 (H*, d, *J* = 0.9, 1H), 7.27 (H6/H6", m, 2H), 7.31 (H8/H8", m, 2H), 7.34 (H2′/H6′, d, *J* = 8.4, 2H), 7.55 (H7/H7", m, 2H), 7.55 (H3′/H5′, d, *J* = 8.4, 2H), 7.85 ppm (H5/H5", dd, *J* = 7.8, 1.5, 2H); ^13^C-NMR (DMSO-d_6_): δ 36.33 (C*), 103.18 (C3/C3"), 115.70 (C8/C8"), 119.07 (C10/C10"), 123.25 (C6/C6"), 124.07 (C5/C5"), 124.58 (CF_3_, q, *J_CF_* = 271.7), 124.77 (C3′/C5′, q, *J_CF_* = 4.2), 125.92 (C4′, q, *J_CF_* = 31.6), 127.44 (C2′/C6′), 131.43 (C7/C7"), 146.86 (C1′), 152.44 (C9/C9"), 164.44 (C2/C2"), 166.95 ppm (C4/C4"); ^19^F-NMR (DMSO-d_6_): δ –60.03 ppm (CF_3_, s); ES-MS *m/z* 481 [M−H]. Elemental analysis. Calc. for C_26_H_15_O_6_F_3_: C 65.01, H 3.15, F 11.86, Found: C 65.11, H 3.25, F 11.97.

*3,3′-(4-Nitrobenzylidene)-bis-[4-hydroxycoumarin]* (**8**) [14]. Yield 84%; mp 236.3 °C; IR (KBr) (ν, cm^−1^): 2858 (CH), 1794 (C=O); 1656 (C=C–C=O), 1600, 1562, 1492, 1454 (C=C); 1390, 1518 (NO_2_); 1196 (OH); ^1^H-NMR (DMSO-d_6_): δ 6.38 (H*, m, 1H), 7.28 (H6/H6", m, 2H), 7.33 (H8/H8", m, 2H), 7.40 (H2′/H6′, m, 2H), 7.57 (H7/H7", m, 2H), 7.86 (H5/H5", dd, *J* = 7.8, 1.5, 2H), 8.08 ppm (H3′/H5′, m, 2H); ^13^C-NMR (DMSO-d_6_): δ 36.70 (C*), 103.13 (C3/C3"), 115.78 (C8/C8"), 118.83 (C10/C10"), 123.17 (C3′/C5′), 123.36 (C6/C6"), 124.07 (C5/C5"), 128.00 (C2′/C6′), 131.60 (C7/C7"), 145.46 (C4′), 150.48 (C1′), 152.44 (C9/C9"), 164.36 (C2/C2"), 166.79 ppm (C4/C4"); ES-MS *m/z* 458 [M−H].

*3,3’-(2-Nitrobenzylidene)-bis-[4-hydroxycoumarin]* (**9**) [11,14]. Yield 73.5%; mp 202 °C; IR (KBr) (ν, cm^−1^): 2874 (CH), 1816 (C=O); 1656 (C=C–C=O), 1604, 1568, 1494, 1452 (C=C), 1524, 1355 (NO_2_), 1199 (OH); ^1^H-NMR (DMSO-d_6_): δ 6.52 (H*, s, 1H), 7.27 (H6/H6", m, 2H), 7.31 (H8/H8", m, 2H), 7.39 (H4′, m, 1H), 7.40 (H6′, m, 1H), 7.53 (H5′, m, 1H), 7.55 (H7/H7", m, 2H), 7.64 (H3′, m, 1H), 7.83 ppm (H5/H5", dd, *J* = 7.8, 1.5, 2H); ^13^C-NMR (DMSO-d_6_): δ 34.18 (C*), 103.07 (C3/C3"), 115.84 (C8/C8"), 118.24 (C10/C10"), 123.37 (C6/C6"), 123.86 (C5/C5"), 123.98 (C3′), 126.96 (C4′), 129.80 (C6′), 131.54 (C7/C7"), 131.84 (C5′), 134.89 (C1′), 149.48 (C2′), 152.35 (C9/C9"), 163.32 (C2/C2"), 165.84 ppm (C4/C4"); ES-MS *m/z* 458 [M−H] (Elemental analysis. Calc. for C_25_H_15_O_8_N: C 65.65, H 3.31, N 3.06, Found: C 65.59, H 3.28, N 3.06.

*3,3’-(4-Methoxybenzylidene)-bis-[4-hydroxycoumarin]* (**10**) [14]. Yield 80%; mp 248.4 °C; IR (KBr) (ν, cm^−1^): 2902 (CH); 1652 (C=C–C=O), 1604, 1564, 1496, 1454 (C=C), 1360 (CH_3_O), 1194 (OH); ^1^H-NMR (CD_2_Cl_2_): δ 3.79 (OCH_3_, s, 3H), 6.03 (H*, s, 1H), 6.85 (H3′/H5′, m, 2H), 7.13 (H2′/H6′, m, 2H), 7.43 (H6/H6", m, 2H), 7.43 (H8/H8", m, 2H), 7.66 (H7/H7", m, 2H), 8.02 (H5/H5", b, 2H), 11.34 (OH, s, 1H) 11.51 ppm (OH, s, 1H); ^13^C-NMR (CD_2_Cl_2_): δ 36.05 (C*), 55.75 (OCH_3_), 104.80/106.32 (C3/C3"), 114.32 (C3′/C5′), 117.02 (C10/C10"), 117.13 (C8/C8"), 124.66 (C5/C5"), 125.40 (C6/C6"), 127.65 (C1′), 128.19 (C2′/C6′), 133.39 (C7/C7"), 152.89/153.01 (C9/C9"), 159.04 (C4′), 164.88/166.20 (C4/C4"), 167.42/169.77 ppm (C2/C2"); ES-MS *m/z* 443 [M−H]. Elemental analysis. Calc. for C_26_H_18_O_7_ (%): C 70.59, H 4.10, Found: C 70.26, H 4.41.

*3,3′-(4-Methyltiobenzylidene)-bis-[4-hydroxycoumarin]* (**11**) [14]. Yield 70%; mp 258.7 °C; IR (KBr) (ν, cm^−1^): 2916 (CH), 2114 (C–S), 1726 (C=O), 1658 (C=C–C=O), 1612, 1566, 1492, 1454 (C=C), 1400 (CH_3_), 1198 (OH); ^1^H-NMR (DMSO-d_6_): δ 2.42 (SCH_3_, s, 3H), 6.28 (H*, s, 1H), 7.08 (H2′/H6′, m, 2H), 7.12 (H3′/H5′, m, 2H), 7.29 (H6/H6", m, 2H), 7.33 (H8/H8", m, 2H), 7.57 (H7/H7", m, 2H), 7.87 ppm (H5/H5", dd, *J* = 7.9, 1.4, 2H); ^13^C-NMR (DMSO-d_6_): δ 15.03 (SCH_3_), 35.63 (C*), 103.80 (C3/C3"), 115.77 (C8/C8"), 118.57 (C10/C10"), 123.43 (C6/C6"), 123.94 (C5/C5"), 126.04 (C3′/C5′), 127.40 (C2′/C6′), 131.56 (C7/C7"), 134.30 (C4′), 137.74 (C1′), 152.29 (C9/C9"), 164.63 (C2/C2"), 166.06 ppm (C4/C4"); ES-MS *m/z* 459 [M−H].

*3,3′-(4-Dimethylaminobenzilidene)-bis-[4-hydroxycoumarin]* (**12**) [11,14]. Yield 65.6%; mp 205.8 °C; IR (KBr) (ν, cm^−1^): 2882 (CH), 1774 (C=O), 1665 (C=C–C=O), 1606, 1568, 1498, 1454 (C=C), 1440 (CH_3_N), 1264 (C–N), 1194 (OH); ^1^H-NMR (DMSO-d_6_): δ 3.13 (CH_3_, s, 6H), 6.28 (H*, s, 1H), 7.24 (H6/H6", m, 2H), 7.24 (H2′/H6′, m, 2H), 7.27 (H8/H8", dd, *J* = 8.1, 1.1, 2H), 7.39 (H3′/H5′, m, 2H), 7.52 (H7/H7", m, 2H), 7.81 ppm (H5/H5", dd, *J* = 7.8, 1.4, 2H); ^13^C-NMR (DMSO-d_6_): δ 35.92 (C*), 45.43 (CH_3_), 103.04 (C3/C3"), 115.58 (C8/C8"), 119.46 (C10/C10"), 119.66 (C3′/C5′), 123.00 (C6/C6"), 124.10 (C5/C5"), 128.15 (C2′/C6′), 131.14 (C7/C7"), 140.82 (C4′), 152.51 (C9/C9"), 164.42 (C2/C2"), 167.60 ppm (C4/C4"); ES-MS *m/z* 456 [M−H]. Elemental analysis. Calc. for C_27_H_21_O_6_N: C 71.20, H 4.65, N 3.08, Found: C 71.23, H 4.61, N 3.11.

*3,3′-(3,4-Dihydroxybenzylidene)-bis-[4-hydroxycoumarin]* (**13**). Yield 70%; mp 227.5 °C; IR (KBr) (ν, cm^−1^): 3568 (OH), 2898 (CH), 1658 (C=O), 1604, 1566, 1498, 1452 (C=C), 1222 (OH); ^1^H-NMR (DMSO-d_6_): δ 6.20 (H*, s, 1H), 6.39 (H6′, m, 1H), 6.55 (H2′, m, 1H), 6.58 (H5′, d, *J*=8.1, 1H), 7.33 (H6/H6", m, 2H), 7.37 (H8/H8", d, *J* = 7.6, 2H), 7.60 (H7/H7", m, 2H), 7.91 ppm (H5/H5", dd, *J* = 7.9, 1.5, 2H); ^13^C-NMR (DMSO-d_6_): δ 35.21 (C*), 104.45 (C3/C3"), 114.22 (C2′), 115.33 (C5′), 115.92 (C8/C8"), 117.38 (C6′), 117.84 (C10/C10"), 123.76 (C6/C6"), 123.86 (C5/C5"), 130.17 (C1′), 131.86 (C7/C7"), 143.19 (C4′), 144.89 (C3′), 152.12 (C9/C9"), 164.76/164.93 ppm (C2/C2"/C4/C4"); ES-MS *m/z* 445 [M−H]. Elemental analysis. Calc. for C_25_H_16_O_8_N: C 67.75, H 3.61, Found: C 67.65, H 3.66.

*3,3′-(2,5-Dimethoxybenzylidene)-bis-[4-hydroxycoumarin]* (**14**). Yield 72%; mp 181.6 °C; IR (KBr) (ν, cm^−1^): 2960 (CH); 1656 (C=C–C=O), 1602, 1568, 1500, 1452 (C=C), 1416 (OCH_3_), 1186 (OH); 1154 (CO); ^1^H-NMR (DMSO-d_6_): δ 3.51 (2′-OCH_3_, s, 3H), 3.62 (5′-OCH_3_, s, 3H), 6.22 (H*, s, 1H), 6.71 (H6′, s, 1H), 6.73 (H4′, m, 1H), 6.81 (H3′, m, 1H), 7.31 (H6/H6", m, 2H), 7.34 (H8/H8", d, *J* = 7.3, 2H), 7.57 (H7/H7", m, 2H), 7.90 ppm (H5/H5", dd, *J* = 7.9, 1.5, 2H); ^13^C-NMR (DMSO-d_6_): δ 33.02 (C*), 55.13 (2′-OCH_3_), 56.13 (5′-OCH_3_), 104.63 (C3/C3"), 110.03 (C4′), 111.84 (C3′), 115.90 (C8/C8"), 115.96 (C6′), 117.77 (C10/C10"), 123.61/123.63 (C5/C5"/C6/C6"), 130.23 (C1′), 131.57 (C7/C7"), 151.63 (C2′), 152.03 (C9/C9"), 152.79 ppm (C5′), 163.96/164.03 (C2/C2"/C4/C4"); ES-MS *m/z* 473 [M−H]. Elemental analysis. Calc. for C_27_H_20_O_8_: C 68.64, H 4.27, Found: C 68.52, H 4.30.

*3,3′-(4-Hydroxy-3-methoxy-5-nitrobenzylidene)-bis-[4-hydroxycoumarin]* (**15**). Yield 70%; mp 199.6 °C; IR (KBr) (ν, cm^−1^): 3274 (OH), 2868 (CH), 1778 (C=O), 1658 (C=C–C=O), 1608, 1566, 1496, 1454 (C=C); 1350, 1634 (NO_2_) 1416 (OCH_3_); 1198 (OH) 1152 (CO); ^1^H-NMR (DMSO-d_6_): δ 3.67 (OCH_3_, s, 3H), 6.23 (H*, t, *J* = 1.0, 1H), 7.01 (H2′, m, 1H), 7.18 (H6′, m, 1H), 7.29 (H6/H6", m, 2H), 7.32 (H8/H8", d, *J* = 7.3, 2H), 7.56 (H7/H7", m, 2H), 7.87 ppm (H5/H5", dd, *J* = 7.8, 1.5, 2H); ^13^C-NMR (DMSO-d_6_): δ 35.81 (C*), 56.58 (OCH_3_), 103.35 (C3/C3"), 113.57 (C6′), 115.80 (C8/C8"), 115.88 (C2′), 118.73 (C10/C10"), 123.40 (C6/C6"), 123.99 (C5/C5"), 131.55 (C7/C7"), 132.16 (C1′), 136.45 (C5′), 140.89 (C4′), 149.16 (C3′), 152.37 (C9/C9"), 164.36 (C2/C2"), 166.45 ppm (C4/C4"); ES-MS *m/z* 504 [M−H]. Elemental analysis. Calc. for C_26_H_17_O_10_N: C 62.03, H 3.40 N 2.78, Found: C 62.33, H 3.34, N 2.89.

*3-[6-Oxo-(1H)-benzopyrano[4,3-b]-(1)10-hydroxybenzopyran-7-yl]-4-hydroxycoumarin* (**16**) [17,18]. Yield 82%; mp 241.7 °C; IR (KBr) (ν, cm^−1^): 2958 (CH), 1616, 1570, 1488, 1456 (C=C), 1230 (C=O); ^1^H-NMR (DMSO-d_6_): δ 5.74 (H*, s, 1H), 7.14 (H5′, m, 1H), 7.20 (H6′, m, 1H), 7.32 (H8, m, 1H), 7.34 (H4′, m, 1H), 7.35 (H3′, m, 1H), 7.36 (H6, m, 1H), 7.46 (H8", m, 1H), 7.49 (H6", m, 1H), 7.60 (H7, m, 1H), 7.71 (H7", dd, *J* = 7.5, 1.7, 1H), 8.03 (H5, m, 1H), 8.11 (H5", dd, *J* = 7.9, 1.4, 1H), 12.21 ppm (OH, b, 1H); ^13^C-NMR (DMSO-d_6_): δ 28.64 (C*), 100.60 (C3), 106.14 (C3"), 113.78 (C10), 116.08/116.22/116.22 (C3′/C8"/C10"), 116.47 (C8), 122.20 (C1′), 122.61 (C5), 123.72 (C5"), 123.97 (C6"), 124.55 (C6), 125.35 (C5′), 128.36 (C4′), 128.62 (C6′), 132.19 (C7"), 132.50 (C7), 149.16 (C2′), 151.97 (C9), 152.17 (C9"), 156.24 (C4), 160.38 (C2), 160.66 ppm (C2"); ES-MS *m/z* 411 [M−H].

*3-[6-Oxo-(1H)-18-bromobenzopyrano[4,3-b]benzopyran-7-yl]-4-hydroxycoumarin* (**17**). Yield 72%; mp 296 °C; IR (KBr) (ν, cm^−1^): 3274 (OH); 2886 (CH); 1772 (C=C–C=O), 1610, 1570, 1558, 1496, (C=C), 1220 (CO), 586 (C–Br); ^1^H-NMR (DMSO-d_6_): δ 5.71 (H*, s, 1H), 7.31 (H6′, d, *J* = 1.8, 1H), 7.32 (H8, m, 1H), 7.34 (H3′, d, *J* = 8.7, 1H), 7.36 (H6, m, 1H), 7.45 (H8", m, 1H), 7.49 (H6", m, 1H), 7.52 (H4′, m, 1H), 7.61 (H7, m, 1H), 7.71 (H7", m, 1H), 8.03 (H5, b, 1H), 8.11 (H5", dd, *J* = 7.9, 1.4, 1H), 12.19 ppm (OH, b, 1H). ES-MS *m/z* 488 [M−H]. Elemental analysis. Calc. for C_25_H_13_O_6_Br: C 61.9, H 2.68, Found: C 61.7, H 2.68.

*3-[6-Oxo-(1H)-16-methoxybenzopyrano[4,3-b]benzopyran-7-yl]-4-hydroxycoumarin* (**18**). Yield 73%; mp 278.3 °C; IR (KBr) (ν, cm^−1^): 3480 (OH); 2906 (CH); 1824 (C=O); 1662 (C=C–C=O), 1620, 1586, 1496, 1460 (C=C), 1440 (OCH_3_), 1188 (OH), 990 (CO); ^1^H-NMR (DMSO-d_6_): δ 3.93 (OCH_3_, s, 3H), 5.72 (H*, s, 1H), 6.75 (H6′, m, 1H), 7.02 (H4′, m, 1H), 7.08 (H5′, m, 1H), 7.33 (H8, m, 1H), 7.36 (H6, m, 1H), 7.45 (H8", d, *J* = 8.2, 1H), 7.51 (H6", m, 1H), 7.60 (H7, m, 1H), 7.70 (H7", dd, *J* = 7.5, 1.7, 1H), 7.99 (H5", dd, *J* = 7.8, 1.5, 1H), 8.06 (H5, m, 1H), 12.26 ppm (OH, b, 1H); ^13^C-NMR (DMSO-d_6_): δ 28.67 (C*), 56.04 (OCH_3_), 100.38 (C3), 104.02 (C3"), 111.18 (C4′), 113.92 (C10), 116.06 (C10"), 116.26 (C8), 116.54 (C8"), 119.66 (C6′), 122.38 (C5"), 122.87 (C1′), 123.8 (C5), 123.99 (C6), 124.65 (C6"), 125.14 (C5′), 132.23 (C7), 132.47 (C7"), 138.64 (C2′), 147.29 (C3′), 151.93 (C9"), 152.16 (C9), 155.94 (C4"), 160.41/160.65 ppm (C2/C2"/C4); ES-MS *m/z* 441 [MH]^+^. Elemental analysis. Calc. for C_26_H_16_O_7_: C 69.77, H 3.66, Found: C 69.85, H 3.84.

*3-[6-Oxo-(1H)-18-nitrobenzopyrano[4,3-b]benzopyran-7-yl]-4-hydroxycoumarin* (**19**). Yield 69%; mp 315.7 °C; IR (KBr) (ν, cm^−1^): 3318 (OH); 2854 (CH), 1818 (C=O); 1675 (C=C–C=O), 1610, 1586, 1498, 1456 (C=C), 1314, 1560 (NO_2_), 1190 (OH); ^1^H-NMR (DMSO-d_6_): δ 5.79 (H*, b, 1H), 7.28 (H8, b, 1H), 7.34 (H6, b, 1H), 7.46 (H8", d, *J* = 8.3, 1H), 7.50 (H6", m, 1H), 7.58 (H7, b, 1H), 7.60 (H3′, d, *J* = 9.0, 1H), 7.71 (H7", m, 1H), 8.01 (H6′, d, *J* = 2.8, 1H), 8.03 (H5, b, 1H), 8.13 (H5", dd, *J* = 7.9, 1.6, 1H), 8.18 (H4′, dd, *J* = 9.0, 2.8, 1H), 12.49 ppm (OH, b, 1H); ES-MS *m/z* 456 [M−H]. Elemental analysis. Calc. for C_25_H_13_O_8_N: C 65.93, H 2.88, N 3.07, Found: C 66.02, H 2.96, N 3.04.

*3-[6-oxo-(1H)-15,17-dimethoxybenzopyrano[4,3-b]benzopyran-7-yl]-4-hydroxycoumarin* (**20**). Yield 68%; mp 287.9 °C; IR (KBr) (ν, cm^−1^): 2940 (CH), 1654 (C=C–C=O), 1596, 1570, 1494, 1456 (C=C), 1438 (OCH_3_), 1276 (CO), 1190 (OH); ^1^H-NMR (DMSO-d_6_): δ 3.67 (6′-OCH_3_, s, 3H), 3.80 (4′-OCH_3_, s, 3H), 5.57 (H*, b, 1H), 6.36 (H5′, d, *J* = 2.4, 1H), 6.59 (H3′, d, *J* = 2.4, 1H), 7.27 (H8, d, *J* = 8.1, 1H), 7.34 (H6, m, 1H), 7.44 (H8", m, 1H), 7.48 (H6", m, 1H), 7.57 (H7, m, 1H), 7.69 (H7", dd, *J* = 7.5, 1.7, 1H), 8.00 (H5, m, 1H), 8.10 (H5", dd, *J* = 7.9, 1.4, 1H), 11.84 ppm (OH, b, 1H); ^13^C-NMR (DMSO-d_6_): δ 24.97 (C*), 55.47 (4′-OCH_3_), 55.95 (6′-OCH_3_), 93.51 (C3′), 95.40 (C5′), 100.79 (C3), 102.59 (C1′), 104.65 (C3"), 113.73 (C10"), 115.97 (C8), 116.32 (C10), 116.44 (C8"), 122.61 (C5"), 123.79 (C5), 123.79 (C6), 124.47 (C6"), 131.80 (C7), 132.43 (C7"), 150.73 (C2′), 151.94 (C9"), 152.11 (C9), 156.27 (C4"), 157.82 (C6′), 159.71 (C4′), 160.31/160.40/160.80 ppm (C2/C2"/C4); ES-MS *m/z* 471 [M−H]. Elemental analysis. Calc. for C_27_H_18_O_8_: C 68.93, H 3.86, Found: C 68.85, H 3.92.

### 4.3. Crystal Structure Determination of ***7***, ***9***, ***16*** and ***18***

Crystals suitable for X-ray single crystal structure study were grown by slow evaporation from dichloromthane for **7** and **18**, toluene for **9** and ethanol solution for **16**. The intensities were collected on an Oxford Diffraction Xcalibur2 diffractometer with a Sapphire 3 CCD detector using graphite-monochromated Mo*K_α_* radiation (*λ* = 0.71073 Å) and *ω* scan-mode at 295 K. *CrysAlisPro* [22] program was used for data collection and processing. The intensities were corrected for absorption using the multi-scan absorption correction method [22]. The crystal structures were solved by direct methods [23] and all non-hydrogen atoms were refined anisotropically by full-matrix least-squares calculations [23] based on *F*^2^ using the programs integrated in *WinGX* [24] program package. The hydrogen atoms attached to the O3 and O3’ atoms in **7** and **9**, O3A and O3B atoms in **16** and O3 atom in **18** were found in a difference Fourier map and are refined with O-H distance restraint of 0.82 Å. All other hydrogen atoms were treated using appropriate riding models, with *SHELXL97* defaults [23]. Fluorine atoms of trifluoromethyl group in **7** are heavily disordered and restraints on anisotropic displacement parameters were therefore used in their refinement. The C29 and C31 atoms of ethanol molecules in **16** are disordered with the site occupancy factors refined to 0.62(3)/0.38(3) and 0.52(3)/0.48(3) ratio, respectively. Details of crystal data, data collection and refinement parameters are given in Table 3. *PLATON* [25] program was used for structure analysis and molecular and crystal structure drawings preparation. CCDC 818091-818094 contain the supplementary crystallographic data for this paper. These data can be obtained free of charge from The Cambridge Crystallographic Data Centre via www.ccdc.cam.ac.uk/data_request/cif.

### 4.4. Virological Assays

The antiviral assays, other than the anti-HIV assays, were based on inhibition of virus-induced cytopathic effect in human lung fibroblast [herpes simplex virus type 1 (HSV-1) [strain KOS], herpes simplex virus type 2 (HSV-2) [strain G], vaccinia virus (VV) and vesicular stomatitis virus (VSV)], African green monkey kidney (Vero, parainfluenza-3, reovirus-1, Sindbis, Coxsackie B4, and Punta Toro virus), human cervix carcinoma (vesicular stomatitis virus, Coxsackie virus B4 and respiratory syncytial virus (RSV)), feline Crandell-Rees kidney (CRFK) (feline herpes virus, feline corona virus (FIPV)) or Madin-Darby canine kidney (MDCK) (influenza A [H1N1; H3N2] and influenza B) cell cultures. Confluent cell cultures in microtiter 96-well plates were inoculated with 100 CCID_50_ of virus (1 CCID_50_ being the virus dose to infect 50% of the cell cultures) in the presence of varying concentrations (100, 40, 8, 1.6 and 0.32 µM) of the test compounds. Viral cytopathicity was recorded as soon as it reached completion in the control virus-infected cell cultures that were not treated with the test compounds. The anti-HIV activity and cytotoxicity of the compounds were evaluated against wild-type HIV-1 strain III_B_ in MT-4 cell culture using the 3-(4,5-dimethylthiazol-2-yl)-2,5-diphenyltetrazolium bromide (MTT) method. MT-4 cells were suspended in culture medium at 1 × 10^5^ cells/mL and infected with HIV at a multiplicity of infection of 0.02. Immediately after viral infection, 100 µL of the cell suspension was placed in each well of a flat-bottomed microtiter tray containing various concentrations of the test compounds. After four days of incubation at 37 °C, the number of viable cells was determined using the MTT method. Compounds were tested in parallel for cytotoxic effects in uninfected MT-4 cells.

## 4. Conclusions

A series of the benzylidene-bis-(4-hydroxycoumarin) derivatives **1**–**15** and fused benzopyranocoumarin derivatives **16**–**20** were synthesized and evaluated for their antiviral activities on a broad panel of DNA and RNA viruses. X-ray crystal structure analysis of 4-trifluoromethylphenyl- and 2-nitrophenyl derivatives **7** and **9** revealed intramolecular hydrogen bonding between hydroxyl and carbonyl oxygen atoms of two 4-hydroxycoumarin moieties resulting in the formation of two eight-membered rings. Accordingly, two 4-hydroxycoumarin moieties in compounds **7** and **9** are anti-disposed. The 4-bromobenzylidene derivative of bis(4-hydroxycoumarin) (**3**) exerted some inhibitory activity against HSV-1 (KOS), HSV-2 (G), vaccinia virus and HSV-1 TK^-^ KOS (ACV^r^) in the range of 9–12 μM at minimum cytotoxic concentration (MCC) greater than 20 *μ*M, whereas the compounds **4**–**6**, **8**, and **20** exhibited rather pronounced anti-Feline Herpes Virus activity (EC_50_ = 5–8.1 μM) but with MCC at only 4-7-fold higher than EC_50_ values.

## Figures and Tables

**Table 1:** Cytotoxicity and antiviral activity of coumarin derivatives **3**, **7**, **8**, **11**, **13**–**15**, **19** and **20** in HEL cell cultures.

Compound	MCC ^a^ (μM)	EC_50_^b^ (μM)				
		HSV-1 (KOS)	HSV-2 (G)	Vaccinia virus	Vesicular stomatitis virus	HSV-1 TK^−^ KOS ACV^r^
**3**	>20	9	9	11	>20	9
**7**	20	>4	>4	>4	>4	>4
**8**	100	>20	>20	>20	>20	>20
**11**	100	>20	>20	>20	>20	>20
**13**	100	>20	>20	>20	>20	>20
**14**	>100	>100	>100	>100	>100	>100
**15**	>100	>100	>100	>100	>100	>100
**19**	>100	50	45	>100	>100	50
**20**	100	>20	>20	>20	>20	>20
Brivudin	>250	0.06	182	3.2	>250	50
Cidofovir	>250	0.9	1.5	7.9	>250	1.5
Acyclovir	>250	0.30	0.35	>250	>250	20
Ganciclovir	>100	0.025	0.030	>100	>100	0.1

^a^ Minimum cytotoxic concentration, as determined by examining the microscopically visible morphological alteration of the cell cultures; ^b^ 50% Effective concentration, or concentration producing 50% inhibition of virus-induced cytopathic effect, as determined by measuring the cell viability with the colorimetric formazan-based MTS assay.

**Table 2:** Cytotoxicity in CRFK cell cultures and anti-Feline Corona Virus (FIPV) and anti-Feline Herpes Virus activity of coumarin derivatives **1**, **4**–**6**, **8**, **9** and **20**.

Compound	Minimum cytotoxic conc. in CRFK cell cultures (CC_50_) ^a^ (μM)	EC_50_^b^ (μM)	
		Feline Corona Virus (FIPV)	Feline Herpes Virus
**1**	43	7.5	9.1
**4**	45	9.1	7.6
**5**	36	7.5	5.0
**6**	39	7.7	8.1
**8**	41	8.5	6.9
**9**	34	8.3	14
**20**	27	6.4	7.1
HHA	> 2	0.34	0.15
UDA	>10	4.2	7.1
Ganciclovir	>100	>100	4.9

^a^ 50% Cytotoxic concentration, as determined by measuring the cell viability by the colorimetric formazan-based MTS assay; ^b^ 50% Effective concentration, or compound concentration producing 50% inhibition of virus-induced cytopathic effect, as determined by measuring the cell viability with the colorimetric formazan-based MTS assay.

**Table 3:** X-ray crystallographic data for compounds **7**, **9**, **16** and **18**.

Compound	7	9	16	18
Formula	C_26_H_15_F_3_O_6_	C_25_H_15_NO_8_	C_54_H_42_O_15_	C_26_H_16_O_7_
Formula weight	480.38	457.38	930.88	440.39
Crystal size [mm]	0.44 × 0.24 × 0.22	0.28 × 0.24 × 0.20	0.62 × 0.35 × 0.29	0.48 × 0.14 × 0.09
Crystal colour, shape	colourless, block	colourless, block	colourless, prism	colourless, prism
Crystal system	monoclinic	monoclinic	monoclinic	triclinic
Space group	*P* 2_1_/*c*	*C* 2/*c*	*P* 2_1_/*c*	*P* $\bar{1}$
Unit cell dimensions				
*a* [Å]	10.4430(4)	34.4429(19)	18.6160(10)	9.4927(4)
*b* [Å]	10.4031(4)	7.5448(3)	15.2918(5)	9.7660(4)
*c* [Å]	20.1801(9)	16.8941(9)	17.7112(10)	11.8741(5)
*α* [°]	90	90	90	66.322(4)
*β* [°]	91.423(3)	112.749(6)	115.484(7)	73.399(4)
*γ* [°]	90	90	90	83.084(3)
*V* [Å^3^]	2191.68(15)	4048.7(3)	4551.3(4)	966.09(7)
*Z*	4	8	4	2
*D*_calc._ [g cm^−3^]	1.456	1.501	1.359	1.514
*μ* [mm^−1^]	0.120	0.114	0.100	0.111
*θ* range [°]	4.14 to 26.00	4.19 to 26.00	4.16 to 26.00	4.21 to 26.00
Collected reflections No.	17378	21957	41605	13789
Independent reflections No. / *R*_int._	4298 / 0.0279	3970 / 0.0229	8906 / 0.0477	3777 / 0.0315
Reflections number *I* ≥ 2σ(*I*)	2649	2694	5836	2341
Data / restraints / parameters	4298 / 20 / 324	3970 / 2 / 315	8906 / 6 / 666	3777 / 1 / 303
Goodness-of-fit on *F*^2^	0.999	1.010	1.020	0.997
*R* [*I* ≥ 2*σ*(*I*)] / *R* [all data]	0.0611 / 0.0926	0.0391 / 0.0587	0.0453 / 0.0878	0.0390 / 0.0707
*wR* [*I* ≥ 2*σ*(*I*)] / *wR* [all data]	0.2004 / 0.2150	0.1103 / 0.1158	0.1039 / 0.1305	0.0954 / 0.1043
Max./min. elect. density [e Å^−3^]	0.543 / −0.425	0.388/ −0.186	0.189 / −0.270	0.216 / −0.174

## Electronic Supplementary Information (ESI)

Tables S1 with hydrogen-bonded geometries, Figures S1-S4 presenting hydrogen bonds for compounds **7**, **9**, **16** and **18**.
